# Adaptation of *Staphylococcus aureus* in a Medium Mimicking a Diabetic Foot Environment

**DOI:** 10.3390/toxins13030230

**Published:** 2021-03-22

**Authors:** Cassandra Pouget, Claude-Alexandre Gustave, Christelle Ngba-Essebe, Frédéric Laurent, Emmanuel Lemichez, Anne Tristan, Albert Sotto, Catherine Dunyach-Rémy, Jean-Philippe Lavigne

**Affiliations:** 1 Virulence Bactérienne et Infections Chroniques, INSERM U1047, Université de Montpellier, 30908 Nîmes, France; cassandra.pouget@gmail.com (C.P.); ngbachristelle@yahoo.fr (C.N.-E.); 2 Centre International de Recherche en Infectiologie, Inserm U1111, CNRS UMR5308, ENS Lyon, Université Claude Bernard Lyon 1, 69365 Lyon, France; claude-alexandre.gustave@outlook.fr (C.-A.G.); frederic.laurent@univ-lyon.fr (F.L.); anne.tristan@chu-lyon.fr (A.T.); 3 Laboratoire de Bactériologie, Institut des Agents Infectieux, Centre National de Référence des Staphylocoques, Hôpital de la Croix-Rousse, Hospices Civils de Lyon, 69365 Lyon, France; 4 Unité des Toxines Bactériennes, UMR CNRS 2001, Institut Pasteur, 75015 Paris, France; emmanuel.lemichez@pasteur.fr; 5 Virulence Bactérienne et Infections Chroniques, INSERM U1047, Université de Montpellier, Department of Infectious and Tropical Diseases, CHU Nîmes, Univ Montpellier, 30908 Nîmes, France; albert.sotto@chu-nimes.fr; 6 Virulence Bactérienne et Infections Chroniques, INSERM U1047, Université de Montpellier, Department of Microbiology and Hospital Hygiene, CHU Nîmes, Univ Montpellier, 30908 Nîmes, France; catherine.remy@chu-nimes.fr

**Keywords:** adaptation, biofilm, diabetic foot infection, EDIN, in vitro model, nematode, Panton–Valentin leukocidin, *Staphylococcus aureus*, virulence

## Abstract

*Staphylococcus aureus* is the most prevalent pathogen isolated from diabetic foot infections (DFIs). The purpose of this study was to evaluate its behavior in an in vitro model mimicking the conditions encountered in DFI. Four clinical *S. aureus* strains were cultivated for 16 weeks in a specific environment based on the wound-like medium biofilm model. The adaptation of isolates was evaluated as follows: by *Caenorhabditis elegans* model (to evaluate virulence); by quantitative Reverse Transcription-Polymerase Chain Reaction (qRT-PCR) (to evaluate expression of the main virulence genes); and by Biofilm Ring test^®^ (to assess the biofilm formation). After 16 weeks, the four *S. aureus* had adapted their metabolism, with the development of small colony variants and the loss of β-hemolysin expression. The in vivo nematode model suggested a decrease of virulence, confirmed by qRT-PCRs, showing a significant decrease of expression of the main staphylococcal virulence genes tested, notably the toxin-encoding genes. An increased expression of genes involved in adhesion and biofilm was noted. Our data based on an in vitro model confirm the impact of environment on the adaptation switch of *S. aureus* to prolonged stress environmental conditions. These results contribute to explore and characterize the virulence of *S. aureus* in chronic wounds.

## 1. Introduction

Diabetes *mellitus* is a public health problem representing a major cause of mortality and morbidity worldwide [1]. This disease affects 442 million adults in the world and represents the seventh leading cause of death (Global report on diabetes: Executive Summary. Available online: https://www.who.int/health-topics/diabetes/publications&publication=who-nmh-nvi-16.3 (accessed on 14 March 2021)). One of the most serious complications of diabetes *mellitus* is foot ulceration due to the triopathy associating ischemia, neuropathy, and arteriopathy [2,3,4]. It is a source of major suffering and financial costs for the patient, but also for the health care professionals and facilities and society. Infection in diabetic foot ulcer (DFU) is particularly frequent and, untreated, ultimately results in lower-limb amputation [5]. Diabetic foot infection (DFI) is estimated to be the most common cause of diabetes-related admission in hospitals. Outcomes in patients presenting with a DFI are poor with a high mortality following this complication (around 15% in the following year) [1,5]. DFIs are polymicrobial and *Staphylococcus aureus* is the most frequent pathogen isolated [6,7]. This Gram-positive coccus is a leading cause of a wide range of diseases, from skin and soft tissue infections (SSTIs) (e.g., impetigo, carbuncles) to life-threatening bacteremia, toxic shock syndrome, endocarditis, and osteomyelitis [6,8]. However, the mechanisms of *S. aureus* pathogenicity and switch from colonization to infection in DFI remain unclear.

*S. aureus* deploys an arsenal of virulence factors to colonize, invade, and destroy tissues and host immune cells [9,10]. These virulence factors include adhesins (called microbial surface components recognizing adhesive matrix molecules (MSCRAMMs), such as fibronectin binding proteins (FnBPs), that bind to different host proteins and are important for tissular colonization [11,12]. The protein A (SpA) promotes immune evasion and protects against host-mediated clearance [10]. *S. aureus* also secretes toxins (e.g., the pore-forming cytotoxin alpha-hemolysin (Hla), which can lead to tissue necrosis. In addition, the superantigens can induce an unregulated polyclonal activation of lymphocyte T cells, leading to a cytokine storm. The regulation of these virulence factors is complex and extensive, but virulence is very closely regulated in *S. aureus*, notably owing to the *agr* gene. This bacterium can also secrete specific toxins such as the Panton–Valentine Leukocidin (PVL), which is a pore-forming toxin [6,13], or the exoenzymes EDINs (EDIN-A, -B, and -C), which inactivate the small GTPase RhoA to promote bacterial dissemination [14,15]. These cytolytic toxins can damage membranes of host cells, leading to cell lysis [16]. Finally, to adapt to the environmental conditions, *S. aureus* is able to survive in a metabolically inactive state while preserving the integrity of the host cell by forming small-colony variants (SCVs). SCVs differ metabolically and phenotypically to ordinary *S. aureus* isolates [17].

During DFI, *S. aureus* has been shown to activate its virulence factors to invade the tissue [18]. However, this infection is chronic and complex, with a different clinical presentation to SSTIs, notably with the mitigated impact of toxinogenic strains [6]. Indeed, *S. aureus* is exposed to various stress conditions in DFU: elevated glucose concentration, decreased temperature, decreased tissue oxygenation, and presence of antibiotics for several weeks [19]. Previous studies have analyzed the impact of antibiotics or glucose on the virulence of *S. aureus* strains during short-term [20,21,22,23,24] or long-term exposure [25,26,27] in a stress environment. These lifestyles involve forming quasi-dormant sub-populations and the presence of persister cells and SCVs [28]. Under prolonged nutrient limitation, the downregulation or loss of *agr* has been reported and seems to play a crucial role [17,28].

The purpose of this study was to evaluate the impact of an in vitro model mimicking the conditions encountered in DFU on the adaptation/switch in the virulence profile of *S. aureus*. This manuscript used the Lubbock wound pathogenic biofilm model [29] firstly adapted by De Leon et al. [30]. This model contains physiological concentrations of blood components, notably utilizing a chopped-meat-based medium supplemented with heparinized plasma and red blood cells. This medium was formulated to represent the conditions of human wounds and better simulate the nutrient environment in the chronic ulcer. In this study, the media formulation was modified by the addition of 10% glucose (allowing a concentration above 150 mg/dL, the limit that defined an uncontrolled diabetes) and two antibiotics at sub-inhibitory concentrations (a condition frequently observed in this pathology due to arteriopathy). The antibiotics used correspond to two of the main molecules used in DFI: vancomycin, a glycopeptide that inhibits cell wall synthesis by binding to the D-Ala-D-Ala terminal of the growing peptide chain during cell wall synthesis; and linezolid, an oxazolidinone that disrupts bacterial growth by inhibiting the initiation process of protein synthesis by binding to a site on the bacterial 23S ribosomal RNA of the 50S subunit. Thus, *S. aureus* virulence was evaluated in this in vitro wound-like medium (WLM) that mimics a diabetic environment.

## 2. Results

### 2.1. Phenotypic Effects on S. aureus after a Prolonged Culture in a Medium Mimicking a DFU Environment

Firstly, we investigated the impact of the WLM supplemented with 10% glucose and/or vancomycin and linezolid tested at 0.5× minimum inhibitory concentration (MIC) on three virulent *S. aureus* strains (NSA739, NSA1077, and NSA7475) isolated from DFI and one colonizing strain (NSA1385) (Table 1). First, the presence/absence of β-hemolysis on blood agar plates and the emergence of SCVs were determined.

#### 2.1.1. Addition of 10% Glucose

The WLM had no impact on the phenotype of the *S. aureus* strains tested after 24 h incubation, with or without the addition of glucose (Supplementary Materials Table S1). However, after 16 weeks in WLM alone, all the bacteria displayed mostly β-hemolytic activity (98–99%), with low level SCV (1–2%) (Table 2). The addition of 10% glucose significantly modified the phenotype of the virulent strains with a clear loss of β-hemolytic production (particularly for NSA739 and NSA1077) and a high level of SCVs compared with the medium without glucose (*p* < 0.01). Interestingly, the colonizing NSA1385 was not significantly affected under any of the conditions tested (Table 2).

#### 2.1.2. Addition of Antibiotics

The addition of sub-MICs of vancomycin (0.5× MIC) and linezolid (0.5× MIC) in WLM supplemented with 10% glucose produced the same observations with a significant modification of the phenotype of the virulent strains (Table 2): a loss of β-hemolytic production (50–95%) and a high level of SCVs (10–30%) compared with WLM alone (*p* < 0.01). These modifications did not affect the colonizing strain NSA1385.

To clearly evaluate the effect of antibiotics, vancomycin and linezolid were tested alone in WLM. Interestingly, in sub-MIC of vancomycin alone, the modifications of *S. aureus* phenotype were variable, with a significant effect only on the β-hemolytic activity on NSA1077 and NSA7475 (*p* < 0.01). No statistical effect was noted for the level of SCV. The addition of linezolid alone significantly altered the β-hemolytic activity of all four tested strains (*p* < 0.01).

### 2.2. Effect of a Prolonged Culture in a Medium Mimicking DFU Environment on S. aureus Virulence

#### 2.2.1. Addition of 10% Glucose

In the *C. elegans* model, all *S. aureus* strains killed the nematodes more rapidly than the avirulent *E. coli* OP50 strain used as nutrient for the nematodes (*p* < 0.001) (Table 3). The LT50 was similar for the three virulent strains (NSA739, NSA1077, and NSA7475), but significantly shorter (*p* < 0.001) for the colonizing strain (NSA1385) (1.7 to 2.3 days ± 0.3 vs. 4.3 ± 0.3, respectively; Table 3). The differences in virulence were not due to differences in the survival and proliferation of strains within the nematode intestine, as the intestine colonization by the strains was not significantly different (Supplementary Materials Table S2).

Infecting *S. aureus* strains pre-cultivated for 24 h in WLM showed significantly decreased virulence compared with the same strains without pre-culture (1.7–2.3 days ± 0.3 vs. 3.5–3.9 ± 0.2, respectively; *p* < 0.001, Table 3). No impact was noted on virulence when these strains were pre-cultivated for 16 weeks (3.5–3.9 ± 0.2 vs. 3.9–4.4 ± 0.2, respectively; *p* = not significant (NS), Table 3). Interestingly, no significant difference of LT50 was noted for the colonizing strain, irrespective of the length of pre-culture (24 h vs. 16 weeks), although an increased nematode lifespan was noted: 4.3 days ± 0.3, without pre-culture; 4.8 ± 0.3, with a 24 h pre-culture; and 5.0 ± 0.4, with a 16-week pre-culture (*p* = NS) (Table 3).

When the infecting *S. aureus* strains were pre-cultivated in WLM supplemented with 10% glucose, their virulence was similar to that observed after pre-culture in WLM alone (3.5–3.9 ± 0.2 vs. 3.5–4.2 ± 0.3, respectively; *p* = NS, Table 3). In contrast, the virulence of the three infecting strains was significantly decreased after a 16-week pre-culture in the glucose supplemented medium compared with medium alone (4.9–5.4 ± 0.4 vs. 3.9–4.4 ± 0.3, respectively; *p* < 0.001, Table 3).

As previously observed, no significant modification of lifespan of the *C. elegans* was observed in the colonizing strain NSA1385 after a short or a long pre-culture in the WLM with or without glucose (24 h: 4.8 ± 0.3 vs. 4.6 ± 0.3 and 16-week: 5.0 ± 0.4 vs. 5.2 ± 0.2, respectively; *p* = NS, Table 3).

#### 2.2.2. Addition of Antibiotics

The addition of vancomycin to the WLM had no effect on the lifespan of nematodes in the presence of *S. aureus*. No significant difference of LT50 of nematodes fed with the different strains was observed when these strains were pre-cultivated for 24 h or 16 weeks in medium plus antibiotic (*p* = NS) (Table 3). However, a trend for increased virulence could be noted for the three infecting *S. aureus* strains pre-cultivated with vancomycin versus without pre-culture (LT50: 3.2–3.7 days ± 0.4 vs. 3.9–4.4 ± 0.3, respectively; *p* = NS, Table 3).

The addition of linezolid also had no significant impact on the virulence of the infecting *S. aureus* pre-cultivated in supplemented WLM for 24 h compared with a pre-culture in WLM medium alone (3.5–4.3 ± 0.3 vs. 3.5–3.9 ± 0.2, respectively; *p* = NS, Table 3). Interestingly, during a long period of pre-culture (16 weeks), a significant increase of the lifespan of the *C. elegans* was observed in two of the three virulent *S. aureus* strains (NSA739 and NSA1077) (4.9–5.4 ± 0.4 vs. 3.9–4.4 ± 0.2, respectively; *p* < 0.001, Table 3). No significant modification of lifespan of the *C. elegans* was observed in the colonizing strain NSA1385 after a short or long pre-culture in the WLM with or without addition of antibiotics (24 h: 4.8 ± 0.3 vs. 5.1 ± 0.2 and 16-week: 5.0 ± 0.4 vs. 5.1 ± 0.3, respectively; *p* = NS, Table 3).

The addition of vancomycin to WLM supplemented with 10% glucose showed the same observation as pre-cultivation without antibiotic. A significant decrease of *S. aureus* virulence was shown for the isolates pre-incubated for 16 weeks (5.0–5.5 ± 0.4 vs. 3.9–4.4 ± 0.3, respectively; *p* < 0.001, Table 3), suggesting a role of glucose alone in this effect.

While the same observation was noted for the long exposure of two of the three isolates (NSA739 and NSA1077) in WLM medium supplemented with 10% glucose and linezolid (4.9–5.5 ± 0.4 vs. 3.9–4.4 ± 0.2, respectively; *p* < 0.001, Table 3), a clear impact of linezolid was also observed after a short exposure (24 h) of the three infecting *S. aureus* to this medium (4.9–5.2 ± 0.4 vs. 3.5–3.9 ± 0.2, respectively; *p* < 0.001, Table 3), suggesting an effect of linezolid on *S. aureus* virulence in the WLM supplemented with glucose.

Finally, no impact of the addition of antibiotics and glucose to WLM medium was noted for the colonizing strain NSA1385 (Table 3).

### 2.3. Effect of a Prolonged Culture in a Medium Mimicking DFU Environment on Kinetics of S. aureus Biofilm Formation

The Biofilm Ring test^®^ was performed to evaluate the effect of WLM with or without the addition of 10% glucose and antibiotics on the capacity of *S. aureus* to form biofilm.

#### 2.3.1. Addition of 10% Glucose

The pre-cultivation of *S. aureus* in WLM supplemented with glucose had no significant effect on biofilm formation for all the studied strains after a short exposure (24 h) (Supplementary Materials Table S3).

**Figure 1:**
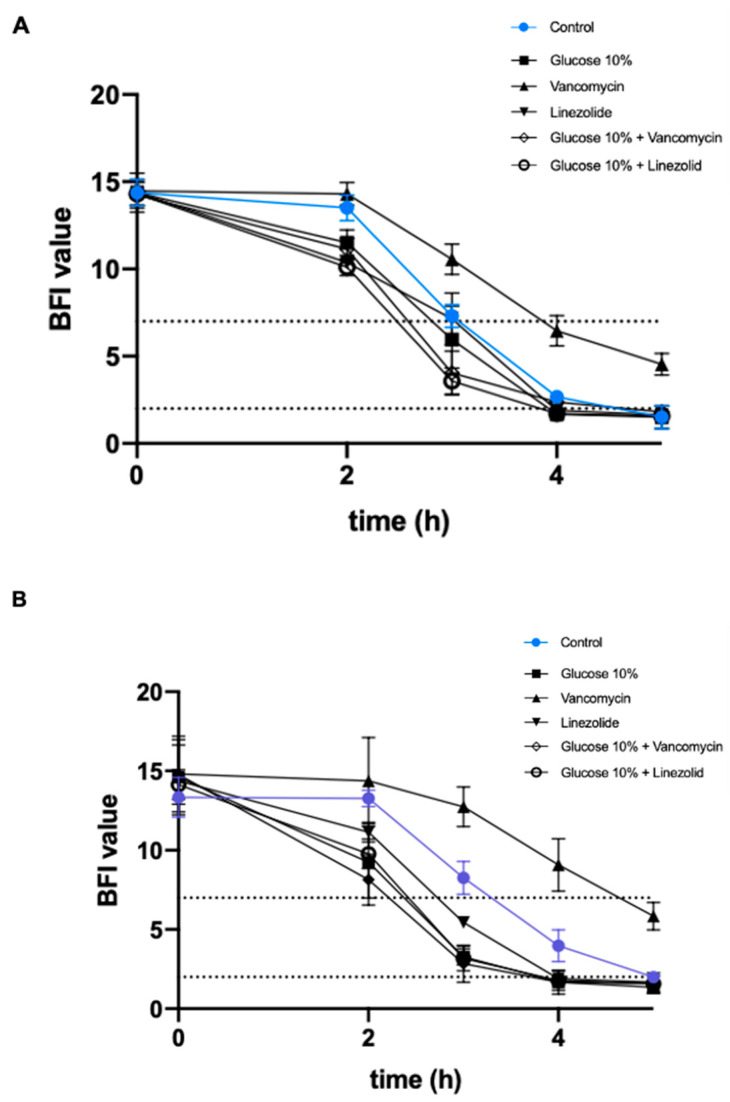

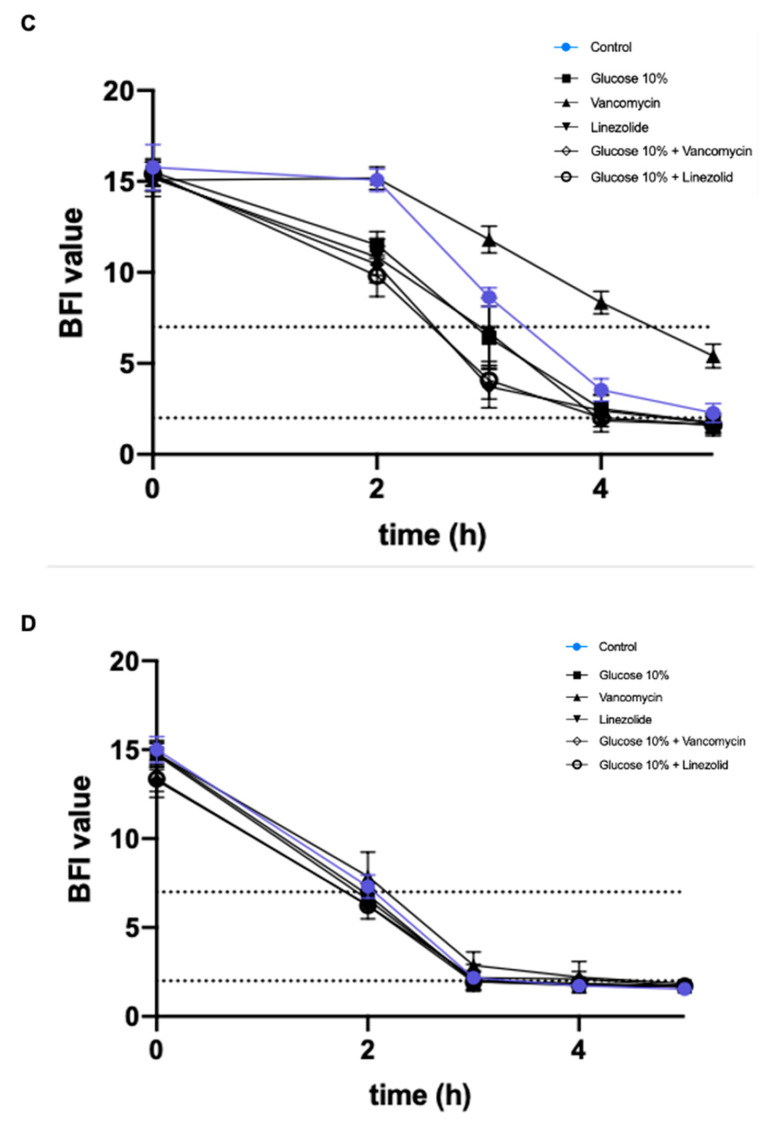
Effects of a pre-culture in a wound-like medium (WLM) and 10% glucose combined with sub-minimum inhibitory concentration (MIC) of vancomycin (0.5× MIC) and linezolid (0.5× MIC) on *S. aureus* biofilm formation after 16 weeks of culture. The kinetics of the early phase of biofilm formation were determined on (**A**) NSA739; (**B**) NSA1077; (**C**) NSA7475; and (**D**) NSA1385 by the BioFilm ring test^®^ (BioFilm Control, France). The control corresponds to the evaluation of biofilm formation of strains in Brain Heart Infusion (BHI) medium alone. Dotted horizontal lines: >7, no biofilm; <2, fixed biofilm. Means ± standard errors of the mean of biofilm indexes (BFIs) for at least three independent replicates are presented. Statistical differences between the different culture conditions at each time were obtained using two-way analysis of variance (ANOVA), followed by Dunnett’s multiple-comparison test.

After a long exposure (16 weeks) of the infecting strains with the WLM supplemented with 10% glucose, all the strains had impeded potential to form biofilm (Figure 1).

For NSA739 and NSA7475, biofilm was completely formed after 5 h of incubation (BFI = 1.5 ± 0.2). When the strain was pre-cultivated in WLM + 10% glucose, we observed a trend of faster formation of biofilm (at 2 and 3 h), but no impact on the biofilm formation at the end-point (5 h).

For NSA1077, the strain pre-cultivated with glucose showed a significantly faster biofilm formation than the strain pre-cultivated in WLM alone at 2, 3, and 4 h, with the beads immobilized after 4 h (BFI = 1.6 ± 0.2) (*p* < 0.001).

For the colonizing strain NSA1385, the addition of glucose had no impact on the kinetics of biofilm formation: the BFI values were similar between all conditions tested.

#### 2.3.2. Addition of Antibiotics

As observed with glucose supplementation, pre-cultivation of *S. aureus* in WLM plus antibiotics (with or without 10% glucose) had no effect on biofilm formation for all the studied strains after a short exposure (24 h) (Supplementary Materials Table S3).

For the three infecting strains NSA739, NSA1077, and NS7475, the kinetics of biofilm formation were slowed in the presence of sub-MIC of vancomycin alone, with a biofilm not constituted after 5 h (*p* < 0.0001) (Figure 1). Interestingly, although the addition of sub-MICs of linezolid had no effect on the biofilm formation of NSA7475 or NSA1077, pre-cultivation of these strains in WLM plus antibiotics and 10% glucose had a significant effect, with the beads immobilized at 3 h (*p* < 0.001).

Antibiotic pressure had no significant impact on the kinetics of biofilm formation for the colonizing strain NSA1385 (Figure 1).

### 2.4. Effect of a Prolonged Culture in a Medium Mimicking DFU Environment on S. aureus Genes’ Expression

**Figure 2:**
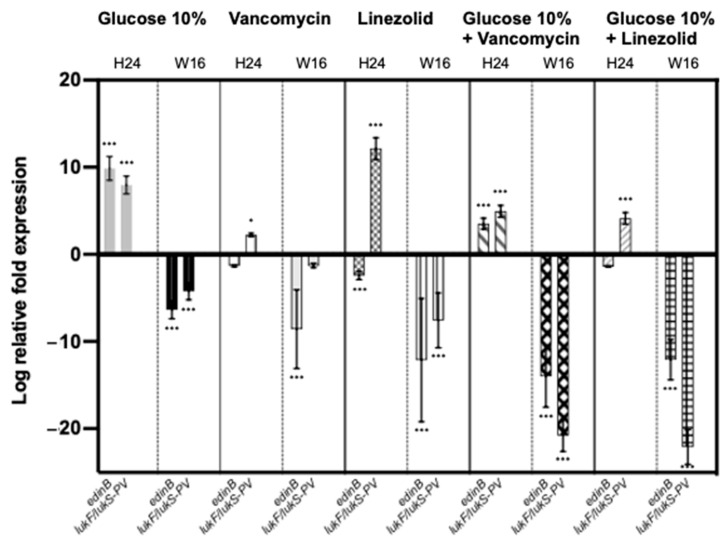
Relative mRNA expression levels of toxinogenic genes of *S. aureus* NSA1077 strain cultivated in WLM supplemented with 10% glucose, vancomycin (0.5× MIC), linezolid (0.5× MIC), 10% glucose + vancomycin (0.5× MIC), and 10% glucose + linezolid (0.5× MIC) after 24 h (H24) and 16 weeks (W16). The log-transformed averages of relative fold changes of NSA1077 in different environmental media compared with NSA1077 in Luria-Bertani (LB) medium for 24 h and 16 weeks are presented. The error bars represent the standard deviations from the three independent RNA preparations. Significant differences from the NSA1077 in LB medium for 24 h and 16 weeks using Dunnett’s test are indicated. *, *p* < 0.01; ***, *p* < 0.001.

**Figure 3:**
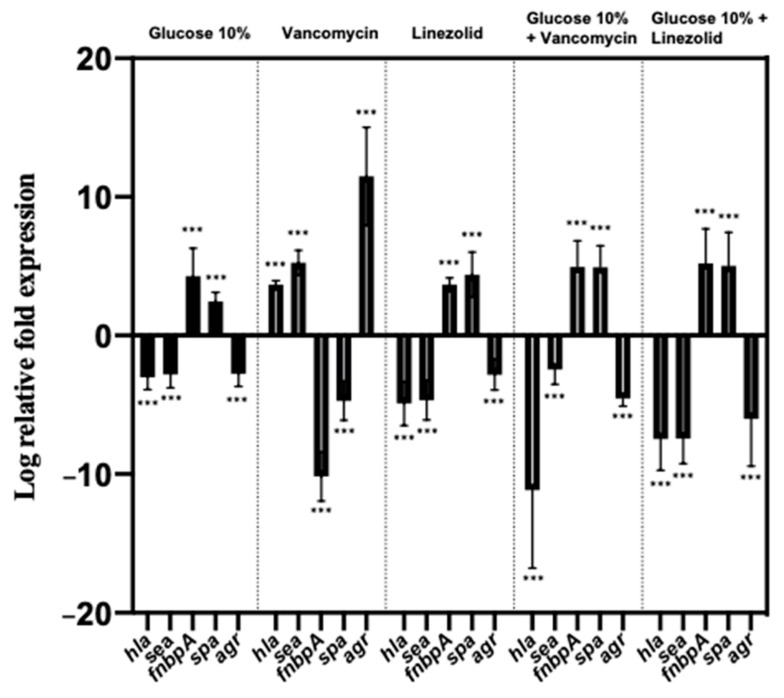
Relative mRNA expression levels of virulence genes and a regulator gene of *S. aureus* NSA739 strain cultivated in WLM supplemented with 10% glucose, vancomycin (0.5× MIC), linezolid (0.5× MIC), 10% glucose + vancomycin (0.5× MIC), and 10% glucose + linezolid (0.5× MIC) after a long exposure (16 weeks). The log-transformed averages of relative fold changes of NSA739 in different environmental media compared with NSA739 in LB medium are presented. The error bars represent the standard deviations from the three independent RNA preparations. Significant differences from the NSA739 in LB medium using Dunnett’s test are indicated. ***, *p* < 0.001.

To assess the impact of WLM on bacteria at the genetic level, the expression levels of some important genes involved in *S. aureus* virulence were measured. Their log relative transcription levels are shown in Supplementary Materials Table S4 for all the strains and Figure 2 and Figure 3 for NSA1077 and NSA739, respectively.

Globally, the expression of studied genes was not significantly affected in strains pre-cultivated in WLM alone (Supplementary Materials Table S4).

#### 2.4.1. Addition of 10% Glucose

After 24 h of exposure to WLM and 10% glucose, *spa* gene, which encodes protein A (a major colonizing factor), was significantly decreased in all the infecting *S. aureus* strains (*p* < 0.05 to *p* < 0.001) (Supplementary Materials Table S4). Moreover, the two toxinogenic *pvl* and *edin*-*B* genes were significantly upregulated (*p* < 0.001) in the two strains harboring these genes: NSA1077 (Figure 2) and NSA7475 (Supplementary Materials Table S4).

No modification in the expression of any tested gene was noted in the colonizing strain NSA1385 (Supplementary Materials Table S4).

The same trend was observed after a long exposure of the infecting strains in WLM supplemented with 10% glucose: *hla*, which encodes the α-hemolysin (another major virulence factor); *sea,* which encodes the staphylococcal enterotoxins (exotoxins with pyrogenicity, superantigenicity, and capacity to enhance lethality of endotoxin); and the global regulator of the staphylococcal virulence, *agr* genes, were significantly down-regulated (*p* < 0.001) (Supplementary Materials Table S4, Figure 3). Moreover, the expressions of the toxinogenic PVL- and EDIN-encoding genes were also significantly decreased (*p* < 0.001) (Supplementary Materials Table S4, Figure 2). In contrast, *spa* and *fnbpA* genes were significantly over-produced when *S. aureus* strains were pre-cultivated in WLM supplemented with 10% glucose (*p* < 0.001) (Supplementary Materials Table S4, Figure 3).

The expression of the studied genes was not modified in any conditions of pre-cultivation for the colonizing strain NSA1385 (Supplementary Materials Table S4).

#### 2.4.2. Addition of Antibiotics

With sub-MIC of vancomycin: Short pre-cultivation of the infecting *S. aureus* strains in WLM plus vancomycin (24 h) did not significantly modify the expression of the virulence genes tested (Supplementary Materials Table S4).

After a long exposure of the infecting strains with the WLM + sub-MIC of vancomycin, *hla*, *sea,* and *agr* genes were significantly up-regulated (*p <* 0.001). In contrast, the expression of *fnbpA* and *spa* genes was significantly decreased (*p <* 0.001) (Supplementary Materials Table S4, Figure 3). Interestingly, although *pvl* gene was not affected by this pre-cultivation, the toxinogenic *edinB* gene was significantly under-expressed (*p <* 0.001) (Supplementary Materials Table S4, Figure 2).

No significant modification in the expression of any studied gene was observed for any pre-cultivating conditions of the colonizing strain NSA1385 (Supplementary Materials Table S4).

With sub-MIC of vancomycin + 10% glucose: The infecting *S. aureus* strains pre-cultivated for 24 h presented a significant increase of *fnbpA*, *spa*, *pvl*, and *edinB* expression (*p <* 0.001) (Supplementary Materials Table S4). Moreover, a decrease of the expression of *hla* gene was noted for NSA1077 (*p <* 0.05).

Contrary to the results with WLM plus vancomycin alone, *hla*, *sea*, and *agr* genes were significantly down-regulated after a long exposure of the infecting strains in WLM + glucose + vancomycin (*p <* 0.001) (Supplementary Materials Table S4, Figure 3). Moreover, the expression of *fnbpA* and *spa* genes was significantly increased (*p <* 0.001) (Supplementary Materials Table S4, Figure 3). Finally, the PVL- and EDIN-encoding genes were significantly under-expressed (*p <* 0.001) (Supplementary Materials Table S4, Figure 2).

After a long exposure of the colonizing strain NSA1385 to the medium, an increased expression of *fnbpA* gene (*p <* 0.001) and a decrease of *hla* gene (*p <* 0.05) (Supplementary Materials Table S4) were observed.

With sub-MIC of linezolid: After a 24 h exposure of the infecting strains in the WLM + linezolid, *hla*, *sea*, and *spa* genes were significantly down-regulated (with the exception of *spa* gene in NSA739) (*p <* 0.05 to *p <* 0.001). In contrast, the expression of *fnbpA* gene was notably increased in NSA739 (*p <* 0.001) (Supplementary Materials Table S2). Interestingly, while PVL-encoding genes were over-produced in this condition, the toxinogenic *edinB* genes were significantly under-expressed (*p <* 0.001) (Supplementary Materials Table S4).

After a 16-week exposure of the infecting strains to WLM + linezolid, *hla*, *sea*, and *agr* genes were significantly down-regulated (*p <* 0.001) (Supplementary Materials Table S4, Figure 3). In contrast, the expressions of *fnbpA* and *spa* genes were significantly increased (*p <* 0.001) (Supplementary Materials Table S4, Figure 3). All the toxinogenic-encoding genes were significantly under-expressed (*p <* 0.001) (Supplementary Materials Table S4, Figure 2).

No significant modification of the expression of the studied genes was observed in any conditions of pre-cultivation for the colonizing strain NSA1385 (Supplementary Materials Table S4).

With sub-MIC of linezolid + 10% glucose: The addition of glucose replicated the results observed with linezolid alone. Two infecting *S. aureus* strains (NSA739 and NSA1077) pre-cultivated in this condition for 24 h presented a significant decrease of *hla* and *sea* genes’ expression (*p <* 0.05), and the infecting strain NSA7475 showed a decrease of *spa* gene (*p <* 0.001) (Supplementary Materials Table S4). Moreover, an increase of the expression of *fnbpA* gene was noted for NSA739 (*p <* 0.001). The PVL-encoding gene was over-expressed in this condition (*p <* 0.001). The only difference concerned the toxinogenic *edinB* gene, which was not significantly modified (Supplementary Materials Table S4).

These different observations were replicated after a long exposure, with a significant decrease of expression of *hla*, *sea*, and *agr* genes (*p <* 0.001) and a significant over-expression of *fnbpA* and *spa* genes (*p <* 0.001) (Supplementary Materials Table S4, Figure 3). The PVL- and EDIN-encoding genes were significantly under-expressed in this condition (*p <* 0.001) (Supplementary Materials Table S4, Figure 2).

Finally, the colonizing strain NSA1385 was significantly affected after a long exposure, with a decrease of *hla* gene expression (*p <* 0.05) and an increase of *fnbpA* gene expression (*p <* 0.001) (Supplementary Materials Table S4).

## 3. Discussion

The host–microbiota interface is often the key point in the development of wound infections. Various studies have described the DFU microbiota to determine the role of microorganisms [33]. Although they have produced interesting results and confirmed that the microbiota is a highly dynamic microbial community that maintains a relationship with the host, better understanding of the complex competitive or synergistic interaction between commensal and/or pathogenic microorganisms is necessary as it could affect the severity and progression of the wound [7]. The virulence capacity of a bacterium has a direct impact on the equilibrium between colonization and infection [34]. However, in chronic wounds, bacteria can adapt their virulence, such as forming a polymicrobial biofilm community [35]. These biofilms may be responsible for the delayed healing of these chronic wounds [36]. Bacterial interactions play an important role in pathogenesis, competing and cooperating in order to support their mutual growth in a specific environment [37]. However, it seems that the behavior of bacteria can also be greatly influenced by the environmental conditions [24]. Here, we evaluated the role of a high glucose concentration and sub-MICs of two antibiotics in a WLM mimicking the conditions encountered in DFU on a series of clinical bacterial strains isolated from this clinical situation. To study bacterial virulence, most studies have previously used in vitro planktonic cultures on rich media or in vivo diabetes animal models (e.g., db/db mice), but without the ability to create genuine wound chronicity [7]. The WLM represents a convenient and reliable model to study bacteria in a relevant environment [29,30].

Using the WLM, we observed that this environment clearly impacts bacterial behavior, and its virulence. Firstly, we observed that a long exposure of *S. aureus* in the medium significantly decreased the bacterial virulence in a *C. elegans* model (Table 3). Moreover, the addition of 10% glucose (a condition similar to that encountered in DFU) more drastically affected the behavior of the infecting bacteria (Table 4): the strains significantly decreased their virulence in the nematodes model (Table 3), in parallel with dramatic under-expression of the virulent genes (and decreased β-hemolytic activity) and an over-expression of genes involved in adhesion and colonization (Figure 3, Supplementary Materials Supplementary Materials Table S4), as observed by Kalan et al. [38]. The bacterial stress induced by the glucose accelerated biofilm formation (Figure 1) and significantly increased SCV phenotypes (Table 2). These observations support previous clinical studies demonstrating that biofilms were implicated in 60 to 80% of chronic wounds versus 6% for acute wounds [39,40]. Bacteria within biofilms evade the host’s natural defenses and are resistant to the host immune defense. Stimulation of the immune system without effectively eradicating the infection causes collateral damage to surrounding tissue and causes chronic inflammation [41]. This persistent chronic inflammation, exacerbated by the diabetic immune context, leads to the production of auto-inflammatory cytokines that aggravate the wound and slow the healing process. The influence of glucose on bacterial virulence has been studied by Regassa et al. [42]. They noted that glucose inhibited the expression of *agr* gene in *S. aureus*. The effect on this regulator gene directly reduced the virulence expression, as observed in our study. Moreover, the influence of glucose on the poly-*N*-acetylglucosamine synthesis, the most common components of biofilm in *S. aureus*, has been previously described [43,44,45]. Glucose stimulates the *gbaAB* operon that regulates biofilm formation through activating the expression of the *ica* operon in *S. aureus*. GbaA is modulated by inducing compounds and facilitates *S. aureus* to adapt to stress via aggregation of biofilm formation [46], as we found here. Interestingly, the response of neutrophils towards *S. aureus* also varies depending on the available glucose. *S. aureus*-mediated NET (neutrophil extracellular trap) release (a process used by neutrophils to kill or trap pathogens) is impaired at a high glucose concentration [47], notably due to an ejection of NET by vesicles outside neutrophils [48]. NET diffusion contributes to extensive tissue damage in the host and could participate in wound chronicity.

Antistaphylococcal regimens must be taken into account in the influence on bacterial virulence, as previously determined [24]. In our study, we observed that vancomycin and linezolid acted differentially on *S. aureus* virulence. This could be explained by the impact of these antibiotics on the expression of regulation of several virulence genes, possibly affecting the expression of the *agr* system. Previous in vitro studies have shown a decreased level of virulence expression upon treatment with ribosomally active antibiotics [24]. Here, the same trend was noted when the bacteria were pre-cultivated in WLM plus linezolid for 24 h. This effect was amplified after a long exposure (16 weeks) to the WLM, especially when combined with a high glucose concentration and sub-MIC of the antibiotic (Figure 2 and Figure 3), applying stress involving a rapid biofilm formation and consistent with the development of a hyper-adhesive phenotype, as previously observed [49]. Studies have shown that linezolid was a potent inhibitor of *hla and sea* genes’ expression in a concentration-dependent manner owing to a positive regulation of *agr* gene [24]. Our results confirmed that these three genes were clearly affected in the same manner by the environmental condition encountered in DFI, while these genes were significantly over-expressed in the presence of sub-MICs of linezolid alone or in combination with glucose (Table 4). The effect of linezolid on *agr* expression is of importance. The link between the dysfunction of this major virulence regulatory system in *S. aureus* and chronic infections has been described [50]. As we noted in our study, this gene plays a crucial role in the evolution to persister cells’ formation and SCVs under antibiotic pressure [50]. The negative impact of linezolid on hemolytic activity has also been noted [51]. Moreover, sub-MICs of linezolid induced a decrease in *spa* expression [21,52]. Although this trend was observed in all the studied strains after a short exposure (24 h) (Supplementary Materials Table S4), the opposite results were observed after a long exposure in the WLM plus linezolid alone or in combination with glucose, suggesting that *S. aureus* clearly modified its virulence in this environment.

The influence of vancomycin on bacterial virulence was totally different compared with linezolid exposure. This antibiotic had limited effects on *S. aureus* virulence after a short exposure, as previously published [24]. In *C. elegans*, no significant modification of bacterial virulence could be noted (Table 3). As seen above, the impact of antibiotics on *agr* expression is crucial. Vancomycin increased the expression of this regulator influencing the virulence by acting on the expression of several virulence genes and by limiting the colonizing behaviour of the bacteria. This trend was confirmed by a huge impact of vancomycin to β-hemolytic activity and the low presence of SCVs even after prolonged exposure in the WLM. Vancomycin-induced upregulation of enterotoxin expression has previously been described during the first 8 h in an in vitro hollow-fiber infection model, followed by a downregulation [53]. Our study confirmed this result. Moreover, previous studies indicated that the effects of vancomycin on *spa* expression were variable [24]. We confirmed that this antibiotic had no relevant impact when used in sub-MIC after a short exposure. In contrast, a significant decrease of *spa* expression was found after a long exposure in the WLM (Table 4).

Finally, the addition of glucose and antistaphylococcal antibiotics demonstrated the main influence of glucose on the bacterial virulence. If the association of glucose plus linezolid impacted more negatively the expressions of *S. aureus* virulence genes, the combination of glucose and vancomycin was clearly influenced by glucose, while a reversed effect of vancomycin alone on genes’ expression level was observed. This is probably due to preferentially interaction between glucose and regulatory genes of *S. aureus* virulence. Further investigations must be developed to confirm this trend.

The PVL is the most studied bi-component leukotoxin produced by *S. aureus* [6]. This toxin confers cytotoxicity on neutrophils and monocytes-macrophages, leading to a high virulence [54]. The PVL-positive strains are responsible for SSTIs, severe necrotizing pneumonia, and aggressive bone and joint infections [55,56,57]. In DFI, the role of PVL in the pathogenicity of *S. aureus* is not clearly established. Firstly, the PVL-producing strains are rarely isolated from this pathology [6]. Its prevalence varies between countries: France (~3%), Algeria, and The Netherlands (~14%) [6]. Classically, the different PVL clones are equally distributed among the various DFI grades. The majority of Grade 1 ulcers where PVL-positive strains were isolated had a rapid clinical amelioration [31]. Moreover, no strains harboring *pvl* gene have been isolated from diabetic foot osteomyelitis (DFOM) [58]. In this study, we explored the impact of environmental conditions on the expression of this gene. We observed that NSA1077 presented a significant decrease of the *pvl* expression after a long exposure in the WLM plus glucose, linezolid, and glucose + linezolid and glucose + vancomycin (Table 4). The antitoxin effect of sub-MICs of linezolid has been previously published [24]. This antibiotic induced concentration-dependent decreases in *pvl* expression and PVL production. Surprisingly, the *pvl* expression was strongly increased after a short exposure to linezolid, in contrast to previous publications. One hypothesis could be the role of the medium used in this study. Interestingly, we observed the same effect when the bacteria were exposed to glucose + linezolid, although to a lesser degree (Figure 2). Sub-MICs of vancomycin have no relevant impact on *pvl* expression [24]. Despite confirming this trend in our study after 24 h exposure to this antibiotic, we observed a significant effect when vancomycin was combined with 10% glucose, with an increase of *pvl* expression after 24 h and a strong decrease of expression after 16 weeks. We suggest that the observed effect was mainly due to glucose.

The same trend was also observed for EDIN toxins. These proteins are members of a group of major bacterial virulence factors targeting host Rho GTPases [59]. Recent findings suggest that EDIN toxins might favor bacterial dissemination in tissues by a hematogenous route, through the induction of large transcellular tunnels in endothelial cells named macroapertures [60,61,62]. In addition, Munro et al. showed that EDIN toxins promote the formation of infection foci in a mouse model of bacteremia [63]. Previous studies on the prevalence of *edin* genes in DFI showed that these genes are rarely isolated in this pathology [64]. EDIN might collaborate with the arsenal of *S. aureus* virulence factors to confer a higher potential for systemic infection [60]. However, bacteremia is a rare complication in DFI [5], possibly because of the impact of environment, as we observed in this work. Altogether, our study corroborates that *S. aureus* can adapt to different environments and infection phases; this adaptation is modulated by tight transcriptional and (post)translational regulation of its virulence factors.

## 4. Conclusions

DFI is a complex environment where multiple bacterial species coexist. The interface between host and bacteria directly affects the healing of the wound. In these chronic ulcers, the virulence of bacteria is influenced by their intrinsic virulence profile and virulent factor equipment, but also by the environmental conditions and the phenotypic switch that these environmental stresses induce. Here, we showed that toxinogenic and non-toxinogenic *S. aureus* decreased their virulence in a WLM mimicking the conditions encountered in chronic wounds or/and required to establish chronic wounds. Our observations deserve to be placed in perspective with the clinical situation and epidemiological data where toxinogenic strains are absent from DFI or DFOM, suggesting that these strains have no clear virulence in this context. Subsequent studies are required to understand how bacteria could adapt their virulence in chronic conditions and to correlate these in vitro data using clinical strains. Such studies are pivotal for better ways to manage of DFU and could help in defining new therapeutics.

## 5. Materials and Methods

### 5.1. Bacterial Strains and Growth Conditions

All bacterial strains used in this study are listed in Table 1.

NSA739, 1077, and 7475 are infecting strains isolated from deep DFI. The strain 1077 was PVL+ and EDIN-B+; the strain 7475 was EDIN-B+. NSA1385 was a colonizing strain isolated from an uninfected ulcer, and NSA739 was isolated from DFI.

The avirulent *Escherichia coli* OP50 was used as a food source and a control for the nematode assays.

We used the in vitro WLM adapted from De Leon et al. that mimics the conditions encountered in the wound [30]. Briefly, the WLM contained 45% Bolton broth, 50% bovine plasma, and 5% laked horse red blood cells. A 1 mL volume of VLM was placed in a 14.5 cm by 1.8 cm glass tube, inoculated with approximatively 10^4^ to 10^5^ CFU of *S. aureus*. The strains were grown in different conditions described in Figure 1. Thus, bacteria were grown at 37 °C with shaking at 220 rpm in WLM supplemented with 10% glucose and/or sub-inhibitory concentration of antibiotics (sub-MICs, 0.5× MIC linezolid and vancomycin). Every day, the optical density was adjusted to OD 0.1. Cultures were maintained for 16 weeks (Figure 1).

The vancomycin and linezolid MICs were determined for each strain by the microdilution method in Mueller–Hinton (MH) as recommended by the European Committee for Antimicrobial Susceptibility Testing (EUCAST) [65]. The impact of sub-MIC exposure for 16 weeks on MICs of the different *S. aureus* strains was evaluated each week. No significant difference could be noted (less than threefold dilution). However, the concentration of antibiotics used in the prolonged cultures was always adapted to these MICs.

Then, 100 µL samples of culture were plated on Columbia agar supplemented with 5% fresh sheep blood (bioMérieux, Marcy l’Etoile, France) at 37 °C each week to assess the loss of beta-hemolysin in each condition.

### 5.2. Nematode Killing Assay

The *C. elegans* infection assay was performed as previously described using the Fer-15 mutant line, which has a temperature sensitive fertility defect [66]. Fer-15 was provided by the Caenorhabditis Genetics Center, which is funded by the NIH National Center for Research Resources (NCRR). To synchronize the growth of nematodes, eggs were collected using the hypochlorite method. Overnight cultures of *E. coli* strains in nematode growth medium (NGM) were harvested, centrifuged, washed once, and suspended in phosphate buffered saline solution (PBS) at pH 7.0 at a concentration of 10^5^ CFU/mL. NGM agar plates were inoculated with 10 µL of bacterial suspension in different conditions (without pre-incubation in WLM, after pre-incubation in WLM alone or with 10% glucose, sub-MICs of vancomycin or linezolid) and incubated at 37 °C for 8–10 h. Plates were brought back to room temperature and seeded with L4 stage *C. elegans* (≈30 per plate). Plates were then incubated at 25 °C and scored each day for live nematode under a stereomicroscope (Leica MS5). A nematode was considered dead when it no longer responded to touch. *C. elegans* that died from being trapped by the wall of the plate were excluded from the analysis.

At least three replicates repeated twice were performed for each selected strain. Lethal time 50% (LT50) corresponded to time (in days) required to kill 50% of the nematodes. The definition of virulence is limited to a narrow definition of virulence and the direct impact of *S. aureus* on the mortality of the worms. Three replicates were performed for each condition.

### 5.3. Feeding Behavior Assays

For occupancy assays, each bacterial strain was cultured overnight at 25 °C in LB media, spotted as a circular lawn on NGM plates, and dried at room temperature for 20 min. Approximately 30 L4 animals were placed in the center of the bacteria lawn. The number of nematodes inside or outside each lawn was counted after 16 h, as previously described [66]. The results were described in percent occupancy corresponding to the number of *C. elegans* in the bacterial lawn/the total number of nematodes. The experiments were performed in triplicate.

The number of bacteria within the *C. elegans* digestive tract was obtained as previously described [67]. Briefly, nematodes were picked at 72 h, and the surface bacteria were removed by washing the nematodes twice in M9 medium containing 25 µg/mL gentamicin. The *C. elegans* were then mechanically disrupted in M9 medium containing 1% Triton X-100 [56]. Serial dilutions of the sample were then plated on LB-agar and the colonies were counted after 24 h. Three replicates were performed for each strain.

### 5.4. Biofilm Formation

To evaluate the biofilm formation, we used the Biofilm ring Test^®^ (BioFilm Control^®^, Saint Beauzire, France) following the manufacturer recommendations [68]. Briefly, standardized bacterial cultures were incubated at 37 °C in 96-well microtiter plates in the presence of magnetic beads. At set time-points, the plates were placed onto a magnetic block and put in the reader. The images of each well before and after magnetic attraction were analyzed with the BioFilm Control software, which gives a biofilm index (BFI). A high BFI value (>7) corresponds to high mobility of the beads under magnetic action (no biofilm), while a low value (<2) corresponds to full immobilization of the beads due to the sessile cells. Three experiments with two repeats were performed per strain and per incubation time.

All the experiments were performed in BHI medium (=control) as recommended by the manufacturer. To study the impact of different conditions tested on biofilm formation, the strains were pre-cultured for 24 h or 16 weeks in WLM with or without 10% glucose and sub-MICs of vancomycin or linezolid. The experiments were done in BHI (the WLM was not adapted to the technology).

### 5.5. qRT-PCR Assays

Virulence genes’ expressions of *S. aureus* were quantified under different stress conditions after 24 h and 16 weeks. The virulence and regulator genes tested were adhesins (*fnbpA, spa*), toxins (*hla, pvl, sea*, *edinB*), and the global regulator *agr* (Supplementary Materials Table S5).

Briefly, overnight *S. aureus* cultures obtained in different media were diluted to an OD_600_ of 0.1 in MH broth and cultured again until an OD_600_ of 0.7 was reached. The OD at 600 nm was then adjusted to 0.75 in Tris buffer (10 mM). A 1.5 mL aliquot of OD-adjusted and washed bacterial suspension was centrifuged at 10,000× *g* for 10 min, and the pellets were treated with lysostaphin (Sigma) at a final concentration of 200 μg/mL. Total RNA from bacteria pellets was extracted as described by the manufacturer using Qiagen RNeasy Mini kit (Qiagen, Courtaboeuf, France) during exponential stages. RNA was treated with the RNase-Free DNase Set (Qiagen, Courtaboeuf, France). Purity and concentration were determined using the NanoDrop^TM^ 2000 spectrophotometer (Fisher Scientific, Pittsburgh, PA, USA). cDNA was synthesized from 1 µg of total RNA for each sample, using the iScript™ Select cDNA Synthesis Kit (Bio-Rad) with random primers according to the manufacturer’s instructions.

Real-time PCR assays were performed in a LightCycler^®^480 device using the LightCycler FastStart DNA Master^PLUS^ SYBRGreen I kit (Roche, Meylan, France) with 100 ng of cDNA and 10 pmol of target primers (Supplementary Materials Table S5) [18,69,70,71,72,73,74]. The specificity of the PCR products was tested by melting point analysis. Amplifications were performed in triplicate from three different RNA preparations. The 2^−∆∆*CT*^ method was used to analyze transcriptional changes in target genes using *gyrB* as the housekeeping control gene (Supplementary Materials Table S5). Error bars indicate the standard deviation (SD) of three independent experiments. Cycle threshold (*Ct*) values of the different target genes were compared with the *Ct*-values of the house-keeping gene (*gyrB*). The normalized relative expressions of the studied genes were obtained for each strain following the equation: 2^−ΔΔ^*^Ct^* (ΔΔCt = (Ct_gene_ − Ct*_gyrB_*)_studied strain in different conditions_ − (Ct_gene_ − Ct*_gyrB_*)_studied strain in LB medium_) [75]. The results obtained for each gene were log-transformed to obtain a fold change difference between strains and conditions used.

### 5.6. Statistical Analysis

Statistics and graphs were prepared using the software package (GraphPad Prism 8.0, GraphPad Software, San Diego, CA, USA).

The comparison between the behavior of *S. aureus* in the in vitro medium alone versus in different conditions was assessed using one-way analysis of variance (ANOVA) followed by Dunnett’s multiple comparisons test.

For the nematode killing assays, differences in survival rates between the different strains were tested by a log-rank (Mantel-cox) test for statistical significance.

Log-transformed data were used for qRT-PCR. The effects of the different additions to the WLM medium on the expression of selected genes and a regulator of *S. aureus* were assessed using one-way analysis of variance (ANOVA), followed by Dunnett’s multiple comparisons test.

The kinetics of biofilm formation were compared by two-way ANOVA, followed by Dunnett’s multiple comparison test.

A *p* < 0.05 was considered to reflect a statistically significant difference.

## Figures and Tables

**Table 1:** *S. aureus* strains used in the study.

Strains	Characteristics	References
**NSA739**	*S. aureus* strain isolated from DFI (Grade 3), PVL-, *edin*-, *agr2*, ST8	[31]
**NSA1385**	*S. aureus* strain isolated from colonized DFU (Grade 1), PVL-, *edin*-, *agr1*, ST8	[31]
**NSA1077**	*S. aureus* strain isolated from DFI (Grade 3), PVL+, *edinB*+, *agr1*, ST152	[32]
**NSA7475**	*S. aureus* strain isolated from DFI (Grade 3), PVL-, *edinB*+, *agr1*, ST25	[32]

DFI, diabetic foot infection; DFU, diabetic foot ulcer; PVL, Panton–Valentine Leukocidin; ST, sequence type.

**Table 2:** Phenotypical modifications of *S. aureus* cultivated in an in vitro wound-like medium (WLM) mimicking the conditions encountered in chronic wounds and with the addition of other stress parameters (high glucose concentration, addition of antibiotics) for 16 weeks. Bold results are statistically significant (*p* < 0.01) compared with WLM alone.

Strains	In Vitro WLM Alone		WLM + 10% Glucose		WLM + Vancomycin		WLM + Linezolid		WLM + 10% Glucose + Vancomycin		WLM + 10% Glucose + Linezolid	
	β-hemol ^1^	SCV ^2^	β-hemol	SCV	β-hemol	SCV	β-hemol	SCV	β-hemol	SCV	β-hemol	SCV
NSA739	99% ± 2	2% ± 2	**12% ± 5**	**22% ± 2**	93% ± 5	2% ± 2	**28% ± 4**	5% ± 2	**5% ± 2**	**30% ± 7**	**13% ± 6**	**18%** **± 7**
NSA1077	98% ± 3	2% ± 2	**5% ± 5**	**11% ± 2**	**91% ± 7**	2% ± 3	**55% ± 5**	**20% ± 5**	**8% ± 4**	**18% ± 5**	**10% ± 10**	**10%** **± 5**
NSA7475	98% ± 2	1% ± 2	**65% ± 3**	**24% ± 2**	**88% ± 7**	4% ± 2	**48% ± 2**	**15% ± 4**	**50% ± 5**	**15% ± 3**	**37% ± 8**	**17%** **± 8**
NSA1385	99% ± 2	0% ± 2	93%± 7	4% ± 2	100% ± 2	0% ± 2	**75% ± 5**	**7% ± 2**	94% ± 2	2% ± 2	91% ± 5	3% ± 3

^1^ β-hemolysis. ^2^ SCV, small colony variant. % corresponds to a mean of counts (±standard deviation) performed on three independent experiments on an average of 200 colonies.

**Table 3:** 50% lethal time (in days) of *Caenorhabditis elegans* infected by different *S. aureus* cultivated in an in vitro wound-like medium (WLM) mimicking the conditions encountered in chronic wounds and with the addition of stress factors (high glucose concentration, sub-minimum inhibitory concentration (MIC) of antibiotics). The results are representative of at least four independent trials for each strain.

Strains	Length of Preculture	Without Pre-Culture in WLM	WLM Alone	WLM + 10% Glucose	WLM + Vancomycin	WLM + Linezolid	WLM + 10% Glucose + Vancomycin	WLM + 10% Glucose + Linezolid
NSA739 24 h	24 h	1.7 ± 0.3	3.5 ± 0.2	3.5 ± 0.3	3.4 ± 0.2	3.8 ± 0.3	3.9 ± 0.2	**5.00 ± 0.4**
NSA739 16-week	16 weeks	NA	3.9 ± 0.2	**4.9 ± 0.2**	3.2 ± 0.3	**4.9 ± 0.2**	**5.0 ± 0.2**	**4.9 ± 0.2**
NSA1077 24 h	24 h	2.2 ± 0.2	3.9 ± 0.2	4.2 ± 0.3	4.1 ± 0.1	4.3 ± 0.2	4.4 ± 0.1	**4.9 ± 0.2**
NSA1077 16-week	16 weeks	NA	4.4 ± 0.1	**5.4 ± 0.4**	3.7 ± 0.2	**5.4 ± 0.4**	**5.5 ± 0.3**	**5.5 ± 0.4**
NSA7475 24 h	24 h	2.3 ± 0.3	3.8 ± 0.2	3.9 ± 0.3	3.7 ± 0.2	3.5 ± 0.2	3.8 ± 0.2	**5.2 ± 0.4**
NSA7475 16-week	16 weeks	NA	4.3 ± 0.3	**5.3 ± 0.4**	3.5 ± 0.2	4.7 ± 0.3	**5.4 ± 0.4**	5.0 ± 0.2
1385 24 h	24 h	4.3 ± 0.3	4.8 ± 0.3	4.6 ± 0.3	4.3 ± 0.4	5.1 ± 0.2	4.8 ± 0.3	4.4 ± 0.2
1385 16-week	16 weeks	NA	5.0 ± 0.4	5.2 ± 0.2	5.1 ± 0.2	5.1 ± 0.3	4.9 ± 0.3	4.9 ± 0.2
OP50 (Control strain)	-	7.7 ± 0.2	NA	NA	NA	NA	NA	NA

In bold, *p* < 0.01 after a pairwise comparison between LT50s (strain in WLM alone vs. others) using a log rank test. NA, not applicable.

**Table 4:** Overview of effect of glucose and sub-MIC antibiotic concentrations on *S. aureus* virulence expression from an in vitro wound-like medium (WLM) mimicking the conditions encountered in chronic wounds.

	Effect on Expression of Virulence Factor ^1^			
	PVL	EDIN	Alpha-Hemolysin	Protein A
Short exposure in WLM added to				
Glucose	↑↑	↑↑	-	↓
Vancomycin	-	-	-	-
Linezolid	↑↑	↓	↓	↓
Long exposure in WLM added to				
Glucose	↓	↓	↓	↑
Vancomycin	-	↓↓	↑	↓
Linezolid	↓↓	↓↓	↓	↑

^1^ ↑, significant increase; ↑↑, >8-fold significant increase; ↓, significant decrease; ↓↓, >8-fold significant decrease; -, no significant effect.

## Data Availability

Data sharing not applicable.

## Supplementary Materials

The following are available online at https://www.mdpi.com/2072-6651/13/3/230/s1, Table S1: Phenotypical modifications of *S. aureus* cultivated in an in vitro wound-like medium (WLM) mimicking the conditions encountered in chronic wounds and with the addition of high glucose concentration and antibiotics during 24 h. Table S2: Evaluation of feeding behavior by measuring bacterial content of *C. elegans* and pathogen avoidance of *S. aureus* cultivated in an in vitro wound-like medium (WLM) mimicking the conditions encountered in chronic wounds and with the addition of high glucose concentration and antibiotics during 16 weeks. Table S3: Effects of a preculture in a WLM and glucose 10% associated or not to sub-MICs of vancomycin (0.5× MIC) and linezolid (0.5× MIC) on *S. aureus* biofilm formation after 24 h of culture. The kinetics of the early phase of biofilm formation were determined on (a) NSA739; (b) NSA1077; (c) NSA7475; and (d) NSA1385 by the BioFilm ring test® (BioFilm Control, France). The results represent the mean of BFIs for at least three independent replicates. Table S4: Relative mRNA expression levels of virulence genes of four *S. aureus* strains cultivated in a WLM added with glucose 10%, vancomycin (0.5× MIC), linezolid (0.5× MIC), glucose 10% + vancomycin (0.5× MIC), and glucose 10% + linezolid (0.5× MIC) after 24 h (H24) and 16 weeks (W16). Table S5: Primers used in the study.
