# Supplementary material for: Adaptation of Staphylococcus aureus in a Medium Mimicking a Diabetic Foot Environment
Source: Toxins (Basel). 2021 Mar 22;13(3):230. doi: 10.3390/toxins13030230 (PMC8005162; doi:10.3390/toxins13030230)
Supplement: Supplementary file 1 [file toxins-13-00230-s001.pdf]

Supplementary Materials: Adaptation of *Staphylococcus aureus* in a Medium Mimicking a Diabetic Foot Environment

Cassandra Pouget, Claude-Alexandre Gustave, Christelle Ngba-Essebe, Frédéric Laurent, Emmanuel Lemichez, Anne Tristan, Albert Sotto, Catherine Dunyach-Rémy and Jean-Philippe Lavigne

Table S1. Phenotypical modifications of *S. aureus* cultivated in an in vitro wound-like medium (WLM) mimicking the conditions encountered in chronic wounds and with the addition of high glucose concentration and antibiotics during 24 h.

	NSA739 Beta-Hemolysis	SCV	NSA1077 Beta-Hemolysis	SCV	NSA7475 Beta-Hemolysis	SCV	NSA1385 Beta-Hemolysis	SCV
WLM alone	100%	0%	100%	0%	100%	0%	100%	0%
WLM + glucose 10%	100%	0%	100%	0%	100%	0%	100%	0%
WLM + vancomycin	100%	0%	100%	0%	100%	0%	100%	0%
WLM + linezolid	100%	0%	100%	0%	100%	0%	100%	0%
WLM + glucose 10% + vancomycin	100%	0%	100%	0%	100%	0%	100%	0%
WLM + glucose 10% + linezolid	100%	0%	100%	0%	100%	0%	100%	0%

Table S2. Evaluation of feeding behavior by measuring bacterial content of *C. elegans* and pathogen avoidance of *S. aureus* cultivated in an in vitro wound-like medium (WLM) mimicking the conditions encountered in chronic wounds and with the addition of high glucose concentration and antibiotics during 16 weeks.

	NSA739 Intestine Survival *	Occupancy Test	NSA1077 Intestine Survival	Occupancy Test	NSA7475 Intestine Survival	Occupancy Test	NSA1385 Intestine Survival	Occupancy Test
Without preculture	5.1 10E5	96 ±3%	6.4 10E5	94 ±5%	4.8 10E5	97 ±3%	5.3 10E5	98 ±2%
WLM alone	4.7 10E5	94 ±5%	5.2 10E5	92 ±4%	4.6 10E5	94 ±5%	5.4 10E5	99 ±2%
WLM + glucose 10%	3.6 10E5	92 ±6%	5.6 10E5	89 ±6%	4.9 10E5	96 ±5%	5.0 10E5	95 ±4%
WLM + vancomycin	5.5 10E5	96 ±2%	5.0 10E5	93 ±3%	4.2 10E5	96 ±4%	5.4 10E5	93 ±5%
WLM + linezolid	6.2 10E5	98 ±2%	4.4 10E5	90 ±5%	4.6 10E5	92 ±6%	4.9 10E5	97 ±2%
WLM + glucose 10% + vancomycin	4.8 10E5	92 ±4%	5.7 10E5	91 ±4%	5.0 10E5	97 ±2%	5.2 10E5	97 ±3%
WLM + glucose 10% + linezolid	3.3 10E5	95 ±3%	5.1 10E5	92 ±6%	4.5 10E5	98 ±2%	5.5 10E5	94 ±4%

Table S3. Effects of a preculture in a WLM and glucose 10% associated or not to sub-MICs of vancomycin (0.5× MIC) and linezolid (0.5× MIC) on *S. aureus* biofilm formation after 24 h of culture. The kinetics of the early phase of biofilm formation were determined on a) NSA739; b) NSA1077; c) NSA7475; d) NSA1385 by the BioFilm ring test® (BioFilm Control, France). The results represent the mean of BFIs for at least three independent replicates.

**NSA739 24 h**	**0 h**	**2 h**	**3 h**	**4 h**	**5 h**	**NSA1077 24 h**	**0 h**	**2 h**	**3 h**	**4 h**	**5 h**
Control BHI medium	15.0	14.7	7.5	2.7	1.8	Control BHI medium	14.4	13.2	8.4	3.9	1.9
Glucose 10%	14.9	14.5	7.9	3.1	1.9	Glucose 10%	14.3	13.8	8.6	4.0	2.0
Vancomycin	14.7	14.6	7.2	2.7	1.9	Vancomycin	14.4	13.2	8.2	3.6	1.9
Linezolid	15.1	14.8	7.7	3.0	2.0	Linezolid	14.2	13.5	8.5	3.7	1.8
Glucose 10% + Vancomycin	15.2	14.9	7.8	2.9	1.9	Glucose 10% + Vancomycin	14.3	13.4	8.3	3.5	1.7
Glucose 10% + Linezolid	14.8	14.6	7.1	2.7	1.8	Glucose 10% + Linezolid	14.1	13.1	8.1	3.6	1.8
**NSA7475 24 h**	0 h	2 h	3 h	4 h	5 h	**NSA1385 24 h**	0 h	2 h	3 h	4 h	5 h
Control BHI medium	15.6	15.1	8.6	3.5	2.3	Control BHI medium	15.1	7.3	2.2	1.7	1.5
Glucose 10%	15.2	14.9	8.3	3.1	2.2	Glucose 10%	15.3	7.5	2.3	1.8	1.4
Vancomycin	15.3	15.0	8.5	3.4	2.1	Vancomycin	15.4	7.4	2.2	1.6	1.6
Linezolid	14.9	14.7	8.4	3.4	2.2	Linezolid	14.8	7.2	2.1	1.7	1.5
Glucose 10% + Vancomycin	15.4	14.9	8.6	3.3	2.2	Glucose 10% + Vancomycin	15.1	7.4	2.4	1.9	1.5
Glucose 10% + Linezolid	14.9	14.6	8.5	3.1	2.1	Glucose 10% + Linezolid	15.3	7.7	2.4	1.7	1.6

Table S4. Relative mRNA expression levels of virulence genes of four *S. aureus* strains cultivated in a WLM added with glucose 10%, vancomycin (0.5× MIC), linezolid (0.5× MIC), glucose 10% + vancomycin (0.5× MIC) and glucose 10% + linezolid (0.5× MIC) after 24 h (H24) and 16 weeks (W16).

		**NSA739 H24**	**NSA739 W16**	**1077 H24**	**1077 W16**	**7475 H24**	**7475 W16**	**1385 H24**	**1385 W16**
WLM alone	*hla*	1.1	−1.88	1.22	−1.92	1.14	−1.78	1.04	−1.25
	sea	1.21	−1.82	1.25	−1.76	1.09	−1.44	1.16	−1.33
	fnbpA	−0.26	1.79	1.04	1.84	1.16	1.56	−0.12	1.21
	spa	−0.34	1.84	−0.22	1.59	1.21	1.66	−0.25	1.52
	agr	1.17	−1.8	1.08	−1.83	1.1	−1.59	1.07	−1.39
	edinB	ND	ND	1.45	−1.66	1.02	1.39	ND	ND
	lukFS-PV	ND	ND	1.55	−1.72	ND	ND	ND	ND
WLM + Glucose 10%	*hla*	1.22	**−3.07**	1.29	**−5.14**	1.08	**−7.59**	1.58	−1.05
	sea	1.27	**−2.89**	1.25	−1.84	1.03	**−2.61**	1.14	−1.67
	fnbpA	−1.96	**4.04**	−1.8	**3.38**	−1.95	**3.08**	−1.13	1.52
	spa	**−2.46**	**2.39**	**−7.66**	**3.44**	**−2.96**	**2.47**	−1.7	1.77
	agr	1.89	**−2.82**	1.91	**−2.58**	1.87	**−2.72**	1.67	−1.42
	edinB	ND	ND	**10.37**	**−6.1**	**8.15**	**−7.41**	ND	ND
	lukFS-PV	ND	ND	**8.12**	**−4.02**	ND	ND	ND	ND
WLM + Vancomycin	*hla*	1.6	**3.72**	1.61	**2.25**	1.07	**2.45**	1.19	1.22
	sea	1.93	**5.14**	1.37	**2.04**	1.23	**3.17**	1.14	1.3
	fnbpA	−1.19	**−9.68**	−1.31	**−3.31**	−1.4	**−4.29**	−1.01	−1.07
	spa	−1.85	**−4.32**	−1.1	**−2.01**	−1.06	**−3.71**	−1.07	−1.66
	agr	1.97	**10.65**	1.95	**2.07**	1.16	**2.41**	1.19	1.08
	edinB	ND	ND	−1.28	**−15.38**	−1.4	**−9.66**	ND	ND
	lukFS-PV	ND	ND	1.88	−1.2	ND	ND	ND	ND
WLM + Linezolid	*hla*	**−2.37**	**−4.57**	**−2.15**	**−3.03**	**−2.03**	**−3.31**	−1.14	−1.05
	sea	**−2.58**	**−4.37**	**−2.05**	**−3.38**	**−2.06**	**−2.49**	−1.42	−1.21
	fnbpA	**2.34**	**3.78**	1.93	**3.39**	1.74	**2.1**	1.49	1.36
	spa	−1.58	**4.25**	**−3.34**	**2.44**	**−3.65**	**8.99**	1.06	1.98
	agr	−1.28	**−2.31**	−1.56	**−2.04**	−1.28	**−2.85**	−1.36	−1.17
	edinB	ND	ND	**−2.26**	**−22.66**	**−3.22**	**−14.38**	ND	ND
	lukFS-PV	ND	ND	**12.51**	**−12.18**	ND	ND	ND	ND
WLM + Glucose + Vancomycin	*hla*	−1.48	**−11.67**	**−2.1**	**−8.96**	−1.5	**−12.52**	−1.12	**−2.01**
	sea	−1.42	**−2.21**	−1.38	**−6.03**	−1.89	**−6.56**	−1.1	−1.67
	fnbpA	**3.47**	**5.34**	**2.9**	**4.08**	**2.56**	**2.93**	1	**3.78**
	spa	**2.84**	**4.61**	**2.19**	**5.71**	**3.33**	**8.31**	1.31	1.99
	agr	−1.97	**−4.49**	−1.46	**−6.37**	−1.23	**−2.88**	−1.27	−1.76
	edinB	ND	ND	**3.6**	**−11.6**	**2.42**	**−13.14**	ND	ND
	lukFS-PV	ND	ND	**4.96**	**−22.96**	ND	ND	ND	ND
WLM + Glucose + Linezolid	*hla*	**−2.13**	**−8.24**	**−2.04**	**−5.09**	−1.5	**−7.26**	−1.1	**−2.08**
	sea	**−2.08**	**−7.09**	**−2.11**	**−2.29**	−1.12	**−3.17**	−1.64	−1.68
	fnbpA	**2.61**	**4.6**	1.24	**2.23**	1.13	**6.82**	1.37	**2.84**
	spa	−1.85	**5.05**	−1.99	**6.25**	**−2.5**	**4.84**	−1.19	1.62
	agr	−1.27	**−4.78**	**−2.43**	**−2.12**	−1.5	**−2.43**	−1.06	−1.9
	edinB	ND	ND	−1.34	**−12.2**	1.2	**−13.07**	ND	ND
	lukFS-PV	ND	ND	**3.98**	**−22.32**	ND	ND	ND	ND

Table S5. Primers used in the study.

Primer Use and Target Function	Target Region	Primer Name	Oligonucleotide Sequence	Tm (°C)	References
**a hemolysin**	*hla*	hla- F	5'- TCCAGTGCAATTGGTAGTCA -3′	55.3	[18]
		hla- R	5'- GGCTCTATGAAAGCAGCAGA-3′	57.3	
**Enterotoxin a**	*sea*	sea- F	5'- ATGGTGCTTATTATGGTTATC -3′	52.0	[69]
		sea- R	5'- CGTTTCCAAAGGTACTGTATT -3′	54.0	
**PVL[a]**	*pvl*	lukS-F	5'- AATAACGTATGGCAGAAATATGGATGT-3′	58.9	[70]
		lukS-R	5'- CAAATGCGTTGTGTATTCTAGATCCT-3′	60.1	
**Edin**	*edinB*	edinB-F	5′- GGTGACGTGAACAAATTATCCGA-3′	58.9	[71]
		edinB-R	5′- ATCTTTCTTTTGTTATCAGAAAGTTTA-3′	54.3	
**MSCCRAMM**	*fnbpA*	fnbpA- F	5'- AAATTGGGAGCAGCATCAGT -3′	55.3	[72]
		fnbpA- R	5'- GCAGCTGAATTCCCATTTTC -3′	55.3	
**Protein A**	*spa*	spa-F	5'- TATGCCTAACTTAAATGCTG -3′	51.1	[73]
		spa- R	5'- TTGGAGCTTGAGAGTCATTA -3′	53.2	
**Accessory gene regulator**	*agrA*	agrA-F	5'- CAAAGAGAAAACATGGTTACCATTATTAA -3′	58.2	[74]
		agrA-R	5'- CTCAAGCACCTCATAAGGATTATCAG -3′	61.6	
**Housekeeping genes**	*gyrB*	gyrB-F	5′- GGTGGCGACTTTGATCTAGC -3′	59.3	[73]
		gyrB-R	5′- TTATACAACGGTGGCTGTGC -3′	57.3	
